# Association Between Early Versus Delayed Outpatient Follow-Up After Hospital Discharge and Short-Term Healthcare Utilization Among ICU Survivors

**DOI:** 10.7759/cureus.107746

**Published:** 2026-04-26

**Authors:** Helen Oletu, Akinyele Oladimeji, Azeberoje Osueni, Emeka Okwuokei

**Affiliations:** 1 Internal Medicine, North Knoxville Medical Center, Powell, USA; 2 Family Medicine, Alberta Health Services, Edmonton, CAN; 3 Gastroenterology and Hepatology, Lagos University Teaching Hospital, Lagos, NGA; 4 Internal Medicine, Hywel Dda University Health Board, Aberystwyth, GBR

**Keywords:** emergency department visits, healthcare utilization, icu survivors, mimic iv, outpatient follow up, readmission

## Abstract

Background: Intensive care unit (ICU) survivors experience high rates of healthcare utilization after hospital discharge, including readmissions and emergency department (ED) visits. Timely outpatient follow-up has been proposed to improve care transitions, but its impact on critically ill populations remains uncertain.

Objective: To assess whether early outpatient follow-up within three days compared with delayed follow-up between seven and 14 days after discharge is associated with differences in 30-day healthcare utilization among adult ICU survivors.

Methods: This retrospective cohort study used data from the Medical Information Mart for Intensive Care IV (MIMIC-IV) database, including linked ED records. Adult ICU survivors discharged from the hospital were included. The exposure was the timing of the first post-discharge outpatient follow-up, defined as the first scheduled outpatient visit after discharge and categorized as early or delayed. ED visits and hospital readmissions were not included in the exposure definition. Outcomes were 30-day hospital readmission and ED visit. Multivariable logistic regression was used to evaluate associations, adjusting for demographic and clinical variables.

Results: A total of 10,123 patients were included. Early follow-up was not associated with differences in 30-day readmission or ED visits compared with delayed follow-up. Higher comorbidity burden and illness severity were associated with increased readmission risk, while exposure timing showed no significant association with either outcome.

Conclusions: Early outpatient follow-up alone may not be associated with differences in short-term healthcare utilization among ICU survivors, although residual confounding and potential biases should be considered. More comprehensive post-discharge care strategies may be needed.

## Introduction

Survivors of intensive care units (ICUs) are associated with high mortality rates and high rates of healthcare utilization after discharge [1]. Recent scientific and technical progress in the field of intensive care medicine has permitted the successful management of critically ill patients in ICUs and has enhanced the chances of survival [2]. Improved survival following discharge from the ICU has been associated with chronic cognitive, psychiatric, and physical impairments among survivors [3]. Post-intensive care syndrome (PICS) is an enormous and prevalent health problem affecting a large percentage of patients who have undergone discharge from the ICU [4]. Such long-term implications of critical illness can affect further healthcare requirements and utilization of health services by these patients [5].

Some of the ways through which follow-up shortly after discharge can enhance the care transition of the patient include prompt medication reconciliation, medication safety, disease management, patient education, and patient-provider communication [6,7]. Patients who had scheduled follow-ups in the next seven days of discharge paradoxically experienced more adverse events after the discharge, as compared to patients whose follow-up was scheduled later [8]. The outpatient benefit of early outpatient follow-up after hospital discharge varies based on the clinical complexity of the patient, as it is an evidence-based process of identifying the optimal time interval for hospital follow-up, depending on the patient's risk segmentation [9].

Apparently, early follow-ups may alleviate symptoms of posttraumatic stress disorder (PTSD) at three to six months post-ICU discharge in survivors of the ICU but do not influence quality of life [10]. Early physician follow-up is effective when it comes to post-discharge adverse events, readmission, emergency department (ED) visits, and mortality reduction [11,12]. ICU follow-up services are comparatively novel in health systems and can potentially serve to address PICS by focusing on unmet health requirements that resulted from the ICU stay [13].

The relationship between post-discharge follow-up and healthcare utilization has been studied in the context of larger populations of patients in the past, and it is possible that follow-up that is provided earlier can decrease readmission and ED visits [14]. Nevertheless, the results have not been consistent, and not many studies directly compared the use of very early follow-up (within three days) to the late follow-up (seven to 14 days) in ICU survivors [15]. Moreover, the current literature also fails to consider the special needs of ICU patients, such as the severity of the illness, increased hospitalization, and the necessity of a multidisciplinary approach [16].

Knowledge on the relationship between outpatient follow-up timing and short-term healthcare use is critical to informing discharge planning and post-acute care plans [17]. By minimizing preventable readmissions and ED visits, the outcome of patients can be enhanced, and the financial load on healthcare systems can be decreased [18,19]. Determining the most successful time within which follow-up care should be administered would aid in better resource utilization and enhancing care transition to survivors of the ICU [20].

The main objective of the study is to assess whether early outpatient follow-up (≤3 days) compared with delayed follow-up (seven to 14 days) after hospital discharge is associated with differences in 30-day hospital readmission and ED visits. The findings of the study will provide important evidence to policymakers and clinical practitioners regarding post-discharge care and ICU survivors.

## Materials and methods

Study design and data source

This study was a retrospective cohort analysis using data from the Medical Information Mart for Intensive Care IV (MIMIC-IV) database, version 3.1, including the linked ED module [21]. The database contains deidentified clinical data from patients admitted to ICUs at a large academic medical center in the United States. Data extraction was performed using structured query language in Google BigQuery (Alphabet Inc., Mountain View, CA, USA). The dataset included information on demographics, hospital admissions, ICU stays, comorbidities, and post-discharge healthcare encounters, including ED visits and hospital readmissions.

Study population

The study population consisted of adult patients with at least one ICU stay during a hospital admission who survived to hospital discharge. Each index admission was defined as a hospitalization with an associated ICU stay and a documented discharge date. Patients who died during the index hospitalization were excluded.

Post-discharge healthcare utilization was assessed using subsequent hospital admissions and ED visits. The exposure variable was defined as the timing of the first post-discharge healthcare contact, identified from recorded outpatient encounters. Healthcare contact included documented outpatient visits and did not include ED visits or hospital readmissions, which were treated as outcomes.

Patients were categorized as early contact if the first outpatient visit occurred within three days of discharge and as delayed contact if it occurred between seven and 14 days. Patients without a recorded outpatient visit within these time windows were excluded from the primary analysis to allow comparison of clearly defined exposure groups.

The choice of time intervals was based on clinically relevant early follow-up periods described in prior literature. However, the exclusion of patients outside these windows and the omission of the four-to-six-day interval may introduce selection bias and should be considered when interpreting the findings. Each hospitalization was treated as an independent observation. The final analytic sample included 10,123 patients.

Variables and measures

The primary exposure was the timing of post-discharge healthcare contact, categorized as early, defined as ≤3 days, and delayed, defined as seven to 14 days. The primary outcomes were hospital readmission within 30 days and ED visit within 30 days of discharge. Outcomes were defined as binary variables based on the presence or absence of an event within the specified time frame. Covariates included demographic and clinical variables selected based on clinical relevance. Age was included as a continuous variable. Gender was categorized as male or female. Race was grouped into Asian, Black, Hispanic, White, and Other individuals. In the MIMIC-IV database, the race category labeled “Other” includes patients whose race is recorded as multiracial, Native American, Pacific Islander, unknown, or not specified in the source data. Insurance type was categorized as Medicaid, Medicare, Private, and Other. Admission type was grouped as emergency, urgent, elective, or other. Clinical severity was measured using the Acute Physiology Score III (APSIII), a component of the Acute Physiology and Chronic Health Evaluation system that quantifies illness severity based on physiological parameters recorded during the ICU stay [22,23]. Comorbidity burden was assessed using the Charlson Comorbidity Index, a validated measure that summarizes overall comorbidity based on the presence and severity of chronic conditions, with higher scores indicating greater disease burden [24]. ICU length of stay was included as a continuous variable. All variables, including APSIII and Charlson Comorbidity Index, were obtained directly from the MIMIC-IV (version 3.1) database using its standardized definitions, consistent with the recommended citation for the database, and no additional external sources were required.

Missing data

Missing data were assessed for all variables included in the analysis. There were no missing values for age, ICU length of stay, APSIII, Charlson Comorbidity Index, readmission outcome, ED visit outcome, exposure variable, race category, admission type, or gender. Insurance type had a small proportion of missing values, with 0.79% of the analytic sample. Given the low proportion of missingness, a complete case analysis approach was used, and observations with missing insurance data were excluded from regression models.

Statistical analysis

Descriptive statistics were used to summarize baseline characteristics by exposure group. Continuous variables were reported as mean and standard deviation, and categorical variables were reported as counts and row percentages. Group comparisons by the exposure variable were conducted using t-tests for continuous variables and chi-square tests for categorical variables. Multivariable logistic regression models were used to evaluate the association between exposure and each outcome, including 30-day readmission and 30-day ED visits. Adjusted odds ratios with 95 percent confidence intervals were reported. All models included age, gender, race category, insurance type, admission type, APSIII, Charlson Comorbidity Index, and ICU length of stay as covariates. Statistical significance was defined using a two-sided p-value less than 0.05. All analyses were performed using Stata version 18 (StataCorp LLC, College Station, TX) [25].

Ethical considerations

The study used a publicly available deidentified dataset. The MIMIC-IV database has been approved for research use with appropriate data use agreements. As the data are deidentified, this study was exempt from institutional review board approval and did not require informed consent.

## Results

Table 1 below presents the baseline characteristics of ICU survivors stratified by timing of post-discharge healthcare contact. In this study, patients in the delayed group were slightly older than those in the early group, with a mean age of 61.35 (16.40) versus 60.13 (17.53), and this difference was statistically significant, p=0.0003. ICU length of stay and APSIII were similar between groups, with no statistically significant differences. The Charlson Comorbidity Index was higher in the delayed group, 5.24 (3.03), compared to 4.88 (3.16), and this difference was statistically significant, p<0.001. Gender distribution was comparable between groups, male 3038 (55.77%) versus 2623 (56.09%) and female 2409 (44.23%) versus 2053 (43.91%), with no significant difference, p=0.746. Race distribution differed across groups, p<0.001, with a higher proportion of early contact among patients classified as Other, 436 (54.91%), compared to delayed, 358 (45.09%). Insurance type showed a modest difference between groups, p=0.047, while admission type was similar between groups, p=0.427.

**Table 1:** Baseline characteristics by the timing of post-discharge healthcare contact –: Intentionally left blank. Values are presented as mean(SD) for continuous variables and n(column percent) for categorical variables. Continuous variables were compared using Student t-tests, and categorical variables were compared using chi-square tests. APSIII refers to Acute Physiology Score III. *Indicates statistically significant p-values (p<0.05). The table was generated and compiled by the authors using Stata version 18 (StataCorp LLC, College Station, TX) [25].

Variable	Delayed (n = 5,447)	Early (n = 4,676)	Test Statistic	p-value
Age, years, mean (SD)	61.35 (16.40)	60.13 (17.53)	t = 3.60	0.0003*
ICU length of stay, mean (SD)	3.71 (5.52)	3.89 (6.00)	t = -1.60	0.110
APSIII, mean (SD)	43.19 (17.88)	42.59 (18.18)	t = 1.65	0.099
Charlson Comorbidity Index, mean (SD)	5.24 (3.03)	4.88 (3.16)	t = 5.87	<0.001*
Gender, n (%)	–	–	χ² = 0.11	0.746
Male	3,038 (55.77%)	2,623 (56.09%)	–	–
Female	2,409 (44.23%)	2,053 (43.91%)	–	–
Race (grouped), n (%)	–	–	χ² = 30.38	<0.001*
White	3,835 (70.41%)	3,198 (68.39%)	–	–
Black	785 (14.41%)	668 (14.29%)	–	–
Hispanic	261 (4.79%)	232 (4.96%)	–	–
Asian	208 (3.82%)	142 (3.04%)	–	–
Other	358 (6.57%)	436 (9.32%)	–	–
Insurance, n (%)	–	–	χ² = 7.97	0.047*
Medicare	2,942 (54.40%)	2,510 (54.15%)	–	–
Medicaid	958 (17.71%)	910 (19.63%)	–	–
Private	1,375 (25.43%)	1,099 (23.71%)	–	–
Other	133 (2.46%)	116 (2.50%)	–	–
Admission type (grouped), n (%)	–	–	χ² = 1.70	0.427
Emergency	3,151 (57.85%)	2,714 (58.04%)	–	–
Urgent	717 (13.16%)	649 (13.88%)	–	–
Elective/Other	1,579 (28.99%)	1,313 (28.08%)	–	–

Table 2 below presents the multivariable logistic regression analysis for 30-day hospital readmission. The results indicate that early contact compared to delayed contact was not associated with 30-day readmission (aOR=1.00,95% CI:0.85-1.17, p=1.000). Age and gender were not significantly associated with readmission. Race was not significantly associated with readmission across categories. Admission type showed a difference, with emergency admission associated with higher odds of readmission (aOR=1.32, 95% CI:1.11-1.57, p=0.002), while urgent admission did not reach statistical significance. A higher APSIII and a higher Charlson Comorbidity Index were both associated with increased odds of readmission, p<0.001 for both. ICU length of stay was not significantly associated with readmission.

**Table 2:** Multivariable logistic regression for 30-day readmission –: Intentionally left blank. Values are adjusted odds ratios with 95 percent confidence intervals derived from multivariable logistic regression. The reference groups were delayed contact for exposure, female for gender, Asian for race, Medicaid for insurance, and elective or other for admission type. APSIII refers to Acute Physiology Score III. *Indicates statistically significant p-values (p<0.05). The table was generated and compiled by the authors using Stata version 18 (StataCorp LLC, College Station, TX) [25].

Variable	Adjusted Odds Ratio (95% CI)	p-value
Exposure (Timing of post-discharge healthcare contact)	–	–
Early(≤3 days) vs Delayed(7–14 days)	1.00 (0.85–1.17)	1.000
Age(years)	1.00 (0.99–1.01)	0.855
Gender	–	–
Male vs Female	0.98 (0.83–1.15)	0.783
Race	–	–
Black vs Asian	0.68 (0.43–1.10)	0.119
Hispanic vs Asian	0.67 (0.39–1.14)	0.138
Other vs Asian	1.59 (0.91–2.76)	0.102
White vs Asian	1.00 (0.64–1.56)	0.999
Insurance	–	–
Medicare vs Medicaid	0.94 (0.74–1.19)	0.595
Other vs Medicaid	0.73 (0.46–1.14)	0.162
Private vs Medicaid	0.93 (0.74–1.18)	0.569
Admission Type	–	–
Emergency vs Elective/Other	1.32 (1.11–1.57)	0.002*
Urgent vs Elective/Other	1.25 (0.96–1.63)	0.099
APSIII (per unit increase)	1.02 (1.01–1.02)	<0.001*
Charlson Comorbidity Index(per unit increase)	1.09 (1.05–1.13)	<0.001*
ICU length of stay(days)	1.01 (0.99–1.03)	0.153

Table 3 below presents the multivariable logistic regression analysis for 30-day ED visits. The results indicate that early contact compared to delayed contact was not associated with 30-day ED visits (aOR=0.96,95% CI:0.89-1.04, p=0.317). Age and gender were not significantly associated with ED visits. Race showed differences, with Black patients having higher odds of ED visits (aOR=1.28,95% CI:1.01-1.62,p=0.045), while patients classified as Other had lower odds (aOR=0.58,95% CI:0.45-0.75, p<0.001). Insurance type was associated with ED visits, with Other and Private insurance showing lower odds compared to Medicaid. Admission type was also associated with emergency admissions, showing lower odds (aOR=0.78,95% CI:0.72-0.86, p<0.001, while urgent admissions showed a modest increase. The APSIII was slightly associated with lower odds of ED visits, p<0.001, while the Charlson Comorbidity Index and ICU length of stay were not significantly associated.

**Table 3:** Multivariable logistic regression for 30-day emergency department visits –: Intentionally left blank. Values are adjusted odds ratios with 95 percent confidence intervals derived from multivariable logistic regression. The reference groups were delayed contact for exposure, female for gender, Asian for race, Medicaid for insurance, and elective or other for admission type. APSIII refers to Acute Physiology Score III. *Indicates statistically significant p-values (p<0.05). The table was generated and compiled by the authors using Stata version 18 (StataCorp LLC, College Station, TX) [25].

Variable	Adjusted Odds Ratio (95% CI)	p-value
Exposure (Timing of post-discharge healthcare contact)	–	–
Early (≤3 days) vs Delayed (7–14 days)	0.96 (0.89–1.04)	0.317
Age (years)	1.00 (1.00–1.01)	0.223
Gender	–	–
Male vs Female	1.06 (0.97–1.14)	0.183
Race	–	–
Black vs Asian	1.28 (1.01–1.62)	0.045*
Hispanic vs Asian	1.12 (0.85–1.49)	0.408
Other vs Asian	0.58 (0.45–0.75)	<0.001*
White vs Asian	0.93 (0.75–1.16)	0.528
Insurance	–	–
Medicare vs Medicaid	1.00 (0.88–1.13)	0.977
Other vs Medicaid	0.74 (0.57–0.98)	0.032*
Private vs Medicaid	0.77 (0.68–0.88)	<0.001*
Admission type	–	–
Emergency vs Elective/Other	0.78 (0.72–0.86)	<0.001*
Urgent vs Elective/Other	1.14 (1.00–1.30)	0.047*
APSIII (per unit increase)	1.00 (0.99–1.00)	<0.001*
Charlson Comorbidity Index (per unit increase)	1.00 (0.99–1.02)	0.829
ICU length of stay (days)	1.00 (0.99–1.01)	0.620

**Figure 1:**
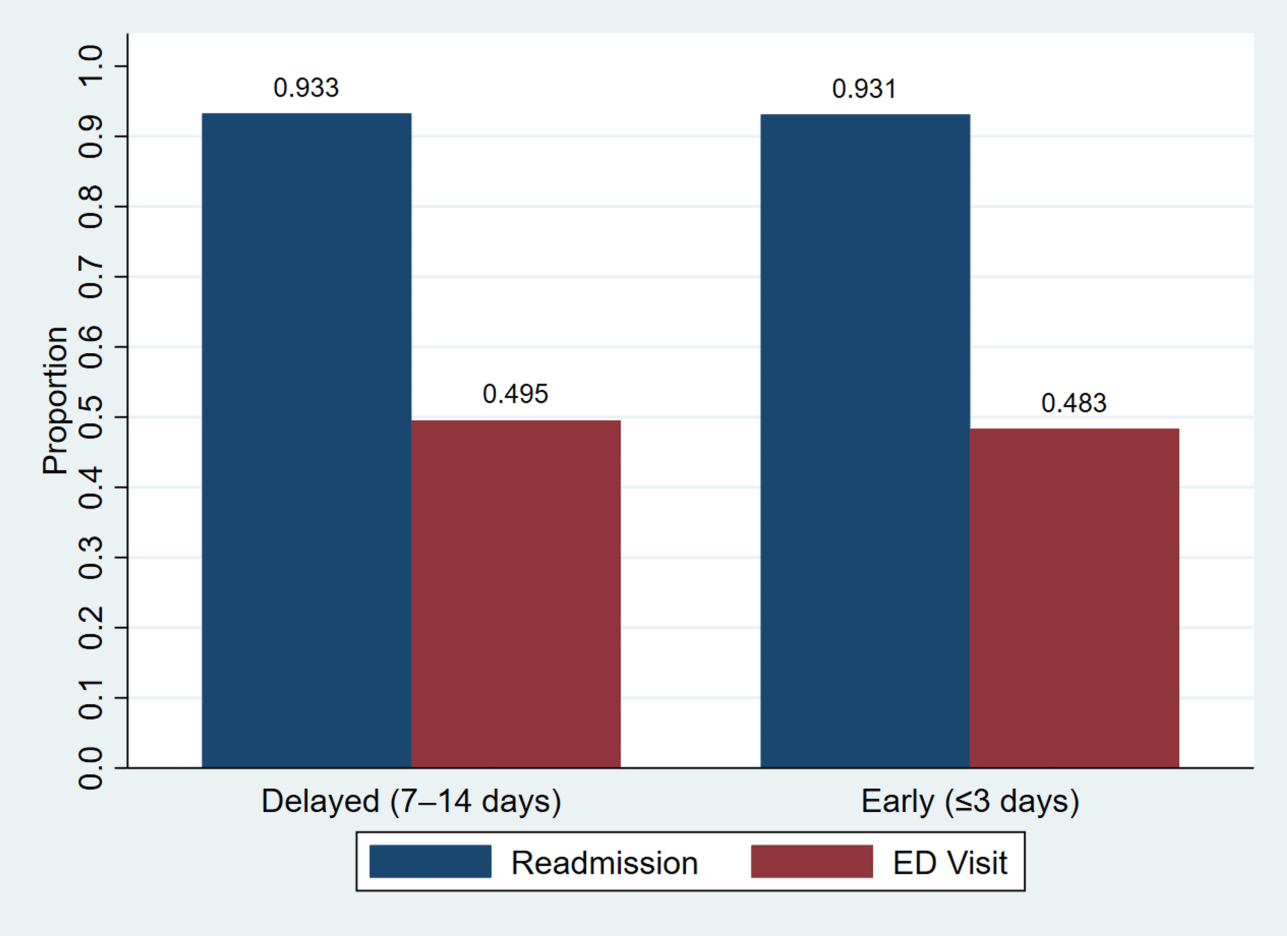
Proportion of 30-day readmission and emergency department visits by exposure group Bars represent mean proportions of outcomes within each exposure group. Readmission refers to hospital readmission within 30 days, and ED visit refers to emergency department visit within 30 days.

Figure 1 illustrates the proportion of 30-day readmission and ED visits by timing of post discharge healthcare contact. The figure shows that the proportion of readmission was similar between the delayed and early groups, 0.933 and 0.931, respectively. The proportion of ED visits was also similar, 0.495 in the delayed group and 0.483 in the early group. Differences between groups appear small, consistent with the regression findings showing no statistically significant association between exposure timing and outcomes.

## Discussion

In this study, the timing of post-discharge healthcare contact was not associated with differences in 30-day hospital readmission or ED visits among ICU survivors. Early follow-up within three days showed similar outcomes compared to delayed follow-up between seven and 14 days. Patient characteristics such as higher comorbidity burden and greater illness severity were associated with increased likelihood of readmission, while demographic and exposure-related factors were not strongly associated with either outcome. These findings suggest that early outpatient contact alone may not be sufficient to influence short-term healthcare utilization in this population.

The findings align with existing literature showing mixed results regarding the benefit of early follow-up after hospital discharge. Previous studies have reported that timely follow-up may reduce adverse events and improve care coordination, particularly in selected populations such as those with chronic conditions [6,8,11]. However, other analyses have found limited or inconsistent effects on readmission outcomes, especially when follow-up is not part of a broader structured care model [7,18]. ICU survivors represent a complex group with high rates of PICS and ongoing healthcare needs, which may not be fully addressed by early outpatient contact alone [2-4]. Prior work has shown that long-term outcomes after critical illness are influenced by multiple factors, including functional status, psychological health, and access to multidisciplinary care services [1,5,14]. This may explain why the timing of follow-up did not show a measurable association with short-term utilization in this cohort. The presence of a higher Charlson Comorbidity Index and elevated APSIII as predictors of readmission is consistent with studies indicating that baseline illness severity and comorbidity burden are key drivers of healthcare utilization after discharge [5,20].

Current guidance in the United States supports timely outpatient follow-up after hospital discharge as part of transitional care strategies. Recommendations emphasize early follow-up to improve continuity of care, medication reconciliation, and identification of post-discharge complications [9]. Transitional care programs that include structured follow-up, patient education, and coordination across care settings have been associated with reductions in readmissions and healthcare utilization [19]. Evidence also suggests that the effectiveness of follow-up depends on the quality and content of the encounter rather than timing alone [12]. In ICU populations, follow-up services such as dedicated recovery clinics have been proposed to address the broad range of physical and psychological sequelae after critical illness [10,13,15]. The present findings are consistent with these recommendations, indicating that timing alone without integrated care interventions may have a limited impact on short-term outcomes.

Several clinical mechanisms may explain the observed results. ICU survivors often experience persistent physical, cognitive, and psychological impairments after discharge, collectively described as PICS [2,3]. These conditions may contribute to ongoing healthcare needs that are not easily modified by a single early outpatient visit. Additionally, early follow-up may identify complications but may not always prevent subsequent acute care use, particularly in patients with a high comorbidity burden. The lack of association between early follow-up and reduced ED visits may also reflect barriers to outpatient care access, variability in care delivery, and differences in patient-level factors such as social support and health literacy [12]. The observed association between admission type and outcomes may indicate differences in underlying illness acuity and healthcare trajectories that extend beyond the timing of follow-up.

Strengths and limitations of the study

This study has several strengths and limitations. The use of a large, detailed clinical database allowed for a comprehensive assessment of patient characteristics and outcomes in a real-world ICU population. The study included objective measures of illness severity and comorbidity burden, which strengthened risk adjustment. However, the analysis did not capture key variables such as outpatient visit type, provider specialty, or the quality and purpose of follow-up encounters. Data on functional status, social determinants of health, and patient-reported outcomes were not available, which may influence post-discharge utilization.

The exclusion of patients without follow-up within the defined time windows and the omission of the intermediate four to six-day period may introduce selection bias and limit generalizability. Additionally, confounding by indication is possible, as patients receiving earlier follow-up may differ systematically in clinical risk or healthcare engagement. The study could not distinguish between planned and unplanned visits or between different types of outpatient encounters, which may affect the interpretation of the findings.

Insurance data had a small proportion of missing values, though this was unlikely to affect results. The observational design limits causal interpretation, and residual confounding may remain. Further, the analysis was based on multivariable regression without the use of advanced methods such as propensity score techniques or sensitivity analyses, which may limit the ability to fully address residual confounding.

Future research should focus on evaluating structured follow-up programs that integrate multidisciplinary care and address the broader needs of ICU survivors.

## Conclusions

This study highlights that early outpatient follow-up within three days after hospital discharge was not associated with differences in short-term healthcare utilization compared to follow-up between seven and 14 days among ICU survivors. Patient-level factors such as comorbidity burden and illness severity were more strongly related to outcomes than the timing of follow-up. These findings suggest that the timing of a single outpatient contact may have a limited influence on readmission and ED use in this population. Clinical care after critical illness may require more comprehensive and coordinated approaches. Future research should focus on structured follow-up models that address functional recovery, multidisciplinary care needs, and access to services to better support ICU survivors after discharge.

## References

[1] Sevin CM, Bloom SL, Jackson JC, Wang L, Ely EW, Stollings JL (2018). Comprehensive care of ICU survivors: development and implementation of an ICU recovery center. J Crit Care.

[2] Vrettou CS, Mantziou V, Vassiliou AG, Orfanos SE, Kotanidou A, Dimopoulou I (2022). Post-intensive care syndrome in survivors from critical illness including COVID-19 patients: a narrative review. Life (Basel).

[3] Ahmad MH, Teo SP (2021). Post-intensive care syndrome. Ann Geriatr Med Res.

[4] Zare-Kaseb A, Sanaie N, Sarmadi S (2026). Prevalence and incidence of post-intensive care syndrome among intensive care unit survivors: a systematic review and meta-analysis. Ann Med.

[5] Hill AD, Fowler RA, Pinto R, Herridge MS, Cuthbertson BH, Scales DC (2016). Long-term outcomes and healthcare utilization following critical illness--a population-based study. Crit Care.

[6] Health Quality Ontario (2017). Effect of early follow-up after hospital discharge on outcomes in patients with heart failure or chronic obstructive pulmonary disease: a systematic review. Ont Health Technol Assess Ser.

[7] Balasubramanian I, Andres EB, Malhotra C (2025). Outpatient follow-up and 30-day readmissions: a systematic review and meta-analysis. JAMA Netw Open.

[8] Tsilimingras D, Ghosh S, Duke A, Zhang L, Carretta H, Schnipper J (2017). The association of post-discharge adverse events with timely follow-up visits after hospital discharge. PLoS One.

[9] Jackson C, Shahsahebi M, Wedlake T, DuBard CA (2015). Timeliness of outpatient follow-up: an evidence-based approach for planning after hospital discharge. Ann Fam Med.

[10] Jensen JF, Thomsen T, Overgaard D, Bestle MH, Christensen D, Egerod I (2015). Impact of follow-up consultations for ICU survivors on post-ICU syndrome: a systematic review and meta-analysis. Intensive Care Med.

[11] Bai JQ, Manokaran T, Meldrum L, Tang KL (2025). Associations between early physician follow-up and post-discharge outcomes: a systematic review and meta-analysis. J Gen Intern Med.

[12] Fu BQ, Zhong CC, Wong CH (2023). Barriers and facilitators to implementing interventions for reducing avoidable hospital readmission: systematic review of qualitative studies. Int J Health Policy Manag.

[13] Schofield-Robinson OJ, Lewis SR, Smith AF, McPeake J, Alderson P (2018). Follow-up services for improving long-term outcomes in intensive care unit (ICU) survivors. Cochrane Database Syst Rev.

[14] Patsaki I, Bachou G, Sidiras G, Nanas S, Routsi C, Karatzanos E (2023). Post hospital discharge functional recovery of critical illness survivors. systematic review. J Crit Care Med (Targu Mures).

[15] Zhang RX, Xu Y, Tian Y, He L, Chu Y (2024). ICU follow-up services and their impact on post-intensive care syndrome: a scoping review protocol. BMJ Open.

[16] Patsaki I, Dimopoulos S (2024). Increasing role of post-intensive care syndrome in quality of life of intensive care unit survivors. World J Crit Care Med.

[17] Pourat N, Chen X, Wu SH, Davis AC (2019). Timely outpatient follow-up is associated with fewer hospital readmissions among patients with behavioral health conditions. J Am Board Fam Med.

[18] Tak HJ, Goldsweig AM, Wilson FA (2021). Association of post-discharge service types and timing with 30-day readmissions, length of stay, and costs. J Gen Intern Med.

[19] Heo M, Taaffe K, Ghadshi A (2023). Effectiveness of transitional care program among high-risk discharged patients: a quasi-experimental study on saving costs, post-discharge readmissions and emergency department visits. Int J Environ Res Public Health.

[20] Lee KK, Yang J, Hernandez AF, Steimle AE, Go AS (2016). Post-discharge follow-up characteristics associated with 30-day readmission after heart failure hospitalization. Med Care.

[21] Johnson A, Bulgarelli L, Pollard T MIMIC-IV (version 3.1). PhysioNet, 2024.

[22] Knaus WA, Wagner DP, Draper EA (1991). The APACHE III prognostic system. Risk prediction of hospital mortality for critically ill hospitalized adults. Chest.

[23] Knaus WA, Draper EA, Wagner DP, Zimmerman JE (1985). APACHE II: A severity of disease classification system. Critical Care Medicine.

[24] Charlson ME, Pompei P, Ales KL, MacKenzie CR (1987). A new method of classifying prognostic comorbidity in longitudinal studies: development and validation. J Chronic Dis.

[25] StataCorp. 2025 (2026). StataCorp. https://www.stata.com/.

